# Correction to “The MAO‐B Inhibitor Selegiline Reduces the Viability of Different Prostate Cancer Cell Lines and Enhances the Effects of Anti‐Androgen and Cytostatic Agents”

**DOI:** 10.1002/prp2.70198

**Published:** 2025-11-28

A. Steib et al., “The MAO‐B Inhibitor Selegiline Reduces the Viability of Different Prostate Cancer Cell Lines and Enhances the Effects of Anti‐Androgen and Cytostatic Agents,” *Pharmacology Research & Perspectives* 13, no. 5 (2025): e70173, https://doi.org/10.1002/prp2.70173.

1. In the author affiliations, “Eötvös Loránd Research Network, Chronic Pain Research Group, Pécs, Hungary” should read “Hungarian Research Network, Chronic Pain Research Group (HUN‐REN PTE), Pécs, Hungary”, and “National Laboratory for Drug Research and Development, Budapest, Hungary” should read “National Laboratory for Drug Research and Development, Magyar tudósok krt. 2. H‐1117 Budapest, Hungary”.
2. In the Funding, “Project no. TKP2021‐EGA‐13 has been implemented with the support provided by the Ministry of Culture and Innovation of Hungary from the National Research, Development and Innovation Fund, financed under the TKP2021 funding scheme.” and “Project no. RRF‐2.3.1‐21‐2022‐00015 has been implemented with the support provided by the European Union.” should be stated.
3. Abbreviations “MAO‐A—monoamine oxidase‐A” and “MAO‐B—monoamine oxidase‐B” should be added.
4. In the Abstract, Materials and Methods, Results and Discussion sections, the concentrations 1, 2, 3, 5 and 10 mM should read 1,000, 2,000, 3,000, 5,000 and 10,000 μM.
5. In the Materials and Methods section, 2.3. MAO activity assay subsection, RT should read room temperature.
6. In the Results section, 3.2. MAO‐B inhibitors significantly reduce the viability of PAC cell lines similarly to MAO‐A blockers subsection, and in the Discussion section, non‐selective MAOI phenelzine should read non‐selective MAO inhibitor phenelzine.
7. The first sentence of the Figure 5. description should be “Network of predicted major proteins and pathways associated with monoamine oxidase‐A and monoamine oxidase‐B in prostate adenocarcinoma.”
8. In the Discussion section, the sentence “Basal SNAI2 mRNA levels were higher in PC‐3, compared to 22Rv1 cells, that can be explained by its metastatic origin, and supported by previous studies also showing elevated levels of SNAI2 mRNA in distant metastases, compared to adjacent lymph node metastases and primary tumor samples.” should read: “Basal SNAI2 mRNA levels were higher in PC‐3 compared to 22Rv1 cells, which can be explained by the metastatic origin of PC‐3. This is supported by previous studies also showing elevated levels of SNAI2 mRNA in distant metastases, compared to adjacent lymph node metastases and primary tumor samples.”

We apologize for this error.

